# Herpesvirus surveillance and discovery in zoo-housed ruminants

**DOI:** 10.1371/journal.pone.0246162

**Published:** 2021-01-28

**Authors:** Teagen G. Partin, Mark D. Schrenzel, Josephine Braun, Carmel L. Witte, Steven V. Kubiski, Justin Lee, Bruce A. Rideout

**Affiliations:** 1 Disease Investigation, San Diego Zoo Global, Escondido, California, United States of America; 2 Hybla Valley Veterinary Hospital, Alexandria, Virginia, United States of America; 3 Department of Microbiology, Immunology, and Pathology, College of Veterinary Medicine and Biomedical Sciences, Colorado State University, Fort Collins, Colorado, United States of America; Loyola University Health System, UNITED STATES

## Abstract

Gammaherpesvirus infections are ubiquitous in captive and free-ranging ruminants and are associated with a variety of clinical diseases ranging from subclinical or mild inflammatory syndromes to fatal diseases such as malignant catarrhal fever. Gammaherpesvirus infections have been fully characterized in only a few ruminant species, and the overall diversity, host range, and biologic effects of most are not known. This study investigated the presence and host distribution of gammaherpesviruses in ruminant species at two facilities, the San Diego Zoo and San Diego Zoo Safari Park. We tested antemortem (blood, nasal or oropharyngeal swabs) or postmortem (internal organs) samples from 715 healthy or diseased ruminants representing 96 species and subspecies, using a consensus-based herpesvirus PCR for a segment of the *DNA polymerase* (*DPOL*) gene. Among the 715 animals tested, 161 (22.5%) were PCR and sequencing positive for herpesvirus, while only 11 (6.83%) of the PCR positive animals showed clinical signs of malignant catarrhal fever. Forty-four *DPOL* genotypes were identified of which only 10 have been reported in GenBank. The data describe viral diversity within species and individuals, identify host ranges of potential new viruses, and address the proclivity and consequences of interspecies transmission during management practices in zoological parks. The discovery of new viruses with wide host ranges and presence of co-infection within individual animals also suggest that the evolutionary processes influencing Gammaherpesvirus diversity are more complex than previously recognized.

## Introduction

The family *Herpesviridae* is a diverse collection of viruses that are divided into three distinct subfamilies—alpha, beta, and gamma—based on genomic content and biology [1–3]. The gammaherpesviruses (GHVs) are members of an expanding subfamily comprised of four genera (*Macavirus*, *Percavirus*, *Lymphocryptovirus*, and *Rhadinovirus*) that infect lymphoid cells and include numerous viruses pathogenic to animals [4]. Macaviruses, in particular, can cause a variety of clinical diseases ranging from subclinical or mild inflammatory syndromes to severe and fatal diseases such as malignant catarrhal fever (MCF) of ruminants [5–19].

Macaviruses are distributed worldwide and diseases associated with these viruses continue to occur in a variety of animal species [11, 20–23]. Prior to our study, the International Committee on the Taxonomy of Viruses (ICTV) officially recognized seven ruminant GHV species [3]. Among those associated with disease, many are classified in the *Macavirus* genus based on expression of the 15A common antigen and sequence data from conserved genes [i.e. Alcelaphine herpesvirus (HV)-1 (AlHV-1), Ovine HV-2 (OvHV-2), Caprine HV-2 (CaHV-2), Bovine HV-6 (BoHV-6), Hippotragine HV, and white-tailed deer MCF virus (WTD-MCFV)].

Certain GHV infections are problematic for captive, zoo, and free-ranging ruminants and have been the focus of numerous investigations aimed at elucidating determinants of their pathogenicity [4, 16, 24–32]. Screening surveys to assess prevalence of members in the *Macavirus* genus have been valuable for improving animal management and established the initial foundation of GHV ecology [33, 34]. Unfortunately, no vaccines are currently available for any macaviruses; therefore, disease prevention relies solely on the segregation of reservoir and susceptible species. Studies describing new viruses have also contributed to characterization of host-pathogen relationships [9, 22]. Yet, much remains unknown about prevalence, diversity, and pathogenic potential of most members.

Although GHVs diverged from other herpesviruses approximately 200 million years ago and continue to drift under varied selection pressures [35, 36], members maintain essential core genes with conserved regions that are the basis of several broad range detection assays [12, 18, 37–40]. Nested PCR methods targeting the *DNA polymerase* (*DPOL)* and *Glycoprotein B* genes are the most widely used discovery tools and have helped identify many of the seven unique ruminant GHVs currently recognized by the ICTV. These assays are often used by diagnostic laboratories and have established the *DPOL* gene as the initial locus for identifying new viruses and taxonomic organization [2, 11, 22, 41]. The current ICTV demarcation criteria for new HV species requires that “(a) their nucleotide sequences differ in a readily assayable and distinctive manner across the entire genome and (b) they occupy different ecological niches by virtue of their distinct epidemiology and pathogenesis or their distinct natural hosts” [42].

To better characterize the genetic diversity of GHVs and their host range, we tested antemortem (blood, nasal or oropharyngeal swabs) or postmortem (internal organs) samples that were collected opportunistically from a large number of healthy or diseased ruminants at the San Diego Zoo and San Diego Zoo Safari Park with a consensus-based herpesvirus PCR for a segment of the *DPOL* gene. The data describe viral diversity within species and individuals, identify potential host ranges of new viruses, and address the proclivity and consequences of viral infection during management practices in zoo animals. The investigation of viral diversity in such a broad survey greatly expands our knowledge of the host-range of new and previously described viruses, and aids in management of zoo-housed ruminants.

## Methods

### Ethics statement

This research was approved by and carried out in strict accordance with San Diego Zoo Global’s Institutional Animal Care and Use Committee’s (IACUC) recommendations. All sampling was performed under IACUC protocol number 231.

### Study population

The study population consists of 715 individuals representing 96 species and subspecies of ruminants housed at San Diego Zoo or San Diego Zoo Safari Park (Table 1). The sampling population included (1) clinically normal animals opportunistically sampled antemortem from 2006–2008 during immobilizations for relocation purposes and annual examinations; (2) animals undergoing routine necropsies between 2006–2008; and (3) select cases presenting with clinical signs compatible with MCF disease and a postmortem diagnosis confirmed by PCR between 1995 and 2010 with available antemortem or postmortem samples. Multiple individuals of a species were sampled to survey viruses for that species in the population. Antemortem samples (n = 1532) included blood (n = 604), nasal swabs (n = 550), and oral or pharyngeal swabs (n = 378). Postmortem samples (n = 1040) included abomasum (n = 1), brain (n = 6), colon (n = 2), fetal tissues (n = 27), heart (n = 1), liver (n = 11), lung (n = 16), nasal tissue (n = 237), placenta (n = 7), retropharyngeal lymph node (n = 247), spleen (n = 238), tongue (n = 3), tonsil (n = 240), oral ulcers (n = 2), and abomasal ulcers (n = 2). All samples were obtained from individuals with no clinical signs of MCF at the time of sampling, with the exception of 11 animals that were diagnosed with MCF: 3 Cape blue duiker (*Philantomba monticola monticola*); 1 Red-flanked duiker (*Cephalophus rufilatus rubidior*); 2 Eastern yellow-backed duiker (*Cephalophus silvicultor silvicultor*); 3 Eastern Bongo (*Tragelaphus eurycerus isaaci*); 1 Domestic goat (*Capra hircus hircus*); and 1 Slender-horned gazelle (*Gazella leptoceros leptoceros*).

**Table 1:** Summary of the sample population, PCR results, and viral genotypes identified by species.

Tested species and subspecies	No. individuals sampled	No. PCR positive individuals	No. sequence positive individuals	Genotype designation in present study^a^
**Family Bovidae**				
Addax (*Addax nasomaculatus*)	20	5	3	22, 27
Addra gazelle (*Gazella dama weidholzi*)	4	0	0	.
Alpine ibex (*Capra ibex*)	8	7	7	7
Angolan roan antelope (*Hippotragus equinus cottoni*)	7	2	1	7
Arabian oryx (*Oryx leucoryx*)	6	2	2	27, 16
Armenian mouflon (*Ovis aries orientalis*)	8	0	0	.
Blackbuck (*Antilope cervicapra rupicapra*)	4	1	1	5
Blesbok (*Damaliscus pygargus phillipsi*)	5	0	0	.
Bontebok (*Damaliscus pygargus dorcas*)	1	0	0	.
Brindled gnu (*Connochaetes taurinus taurinus*)	1	0	0	.
Cape blue duiker (*Philantomba monticola monticola*)	6	5	5	1, 2, 11, 41
Cape buffalo (*Syncerus caffer caffer*)	2	0	0	.
Cavendish's dik dik (*Madoqua kirkii cavendishi*)	2	2	2	39
Chinese bharal (*Pseudois nayaur*)	22	0	0	.
Chinese goral (*Naemorhedus griseus griseus*)	1	0	0	.
Cretan wild goat (*Capra hircus cretica*)	20	2	1	5
Cuvier's gazelle (*Gazella cuvieri*)	9	1	1	16
Desert bighorn (*Ovis canadensis nelsoni*)	6	2	2	16
Domestic cattle (*Bos taurus*)	9	3	3	10, 32
Domestic goat (*Capra hircus hircus*)	3	2	2	38, 44
Domestic sheep (*Ovis aries aries*)	1	0	0	.
East African eland (T*aurotragus oryx pattersonianus*)	13	7	1	7
East African sitatunga (*Tragelaphus spekii spekii*)	16	5	3	23
East Caucasian tur (*Capra caucasica cylindricornis*)	28	8	3	3
Eastern bongo (*Tragelaphus eurycerus isaaci*)	4	3	3	3
Eastern giant eland (*Taurotragus derbianus gigas*)	6	3	3	3, 19
Eastern white-bearded gnu (*Connochaetes taurinus albojubatus*)	11	0	0	.
Eastern yellow-backed duiker (*Cephalophus silvicultor silvicultor*)	6	5	2	4, 6, 9
Ellipsen waterbuck (*Kobus ellipsiprymnus ellipsiprymnus*)	11	10	7	10, 34, 35
Fringe-eared oryx (*Oryx beisa callotis*)	10	2	0	.
Gambian Maxwell's duiker (*Philantomba maxwellii maxwellii*)	1	1	1	4
Gemsbok (*Oryx gazella*)	6	2	2	31
Grant's gazelle (*Nanger granti granti*)	17	0	0	.
Himalayan tahr (*Hemitragus jemlahicus jemlahicus*)	4	0	0	.
Indian gaur (*Bos frontalis gaurus*)	2	0	0	.
Japanese serow (*Capricornis crispus*)	2	0	0	.
Javan banteng (*Bos javanicus javanicus*)	4	2	1	4
Jimela topi (*Damaliscus korrigum jimela*)	9	4	4	15, 20
Kenya impala (*Aepyceros melampus rendilis*)	9	4	4	26
Lake Victoria Defassa waterbuck (*Kobus ellipsiprymnus adolfifriderici*)	2	1	0	.
Lesser kudu (*Tragelaphus imberbis*)	4	0	0	.
Lowland anoa (*Bubalus depressicornis*)	7	0	0	.
Lowland nyala (*Tragelaphus angasii*)	7	0	0	.
Nile lechwe (*Kobus megaceros*)	12	5	5	22, 31
Nubian ibex (*Capra nubiana*)	3	1	1	28
Nubian soemmerring's gazelle (*Nanger soemmerringii soemmerringii*)	8	0	0	.
Persian goitered gazelle (*Gazella subgutturosa subgutturosa*)	17	4	2	2, 29
Red-flanked duiker (*Cephalophus rufilatus rubidior*)	6	5	5	11, 12
Rocky Mountain bighorn (*Ovis canadensis canadensis*)	1	0	0	.
Royal antelope (*Neotragus pygmaeus*)	4	1	0	.
Sand gazelle (*Gazella subgutturosa marica*)	4	1	1	25
Scimitar-horned oryx (*Oryx dammah*)	19	3	2	10
Siberian ibex (*Capra sibirica*)	1	0	0	.
Sichuan takin (*Budorcas taxicolor tibetana*)	6	0	0	.
Slender-horned gazelle (*Gazella leptoceros leptoceros*)	4	3	3	15
South African greater kudu (*Tragelaphus strepsiceros strepsiceros*)	3	3	2	23
South African sable antelope (*Hippotragus niger niger*)	8	0	0	.
South African springbok (*Antidorcas marsupialis marsupialis*)	14	7	7	23
Southern gerenuk (*Litocranius walleri walleri*)	6	2	2	3, 19
Southern steenbok (*Raphicerus campestris campestris*)	7	2	2	42
Spanish ibex (*Capra pyrenaica*)	1	0	0	.
Speke's gazelle (*Gazella spekei*)	6	1	1	40
Sudan barbary sheep (*Ammotragus lervia blainei*)	6	0	0	.
Sudan red-fronted gazelle (*Eudorcas rufifrons laevipes*)	9	3	3	4, 8, 30, 33
Thomson's gazelle (*Eudorcas thomsonii thomsonii*)	6	0	0	.
Transcaspian urial (*Ovis aries arkal*)	6	0	0	.
Turkomen markhor (*Capra falconeri heptneri*)	8	0	0	.
Uganda kob (*Kobus kob thomasi*)	15	4	3	4, 22
Vaal rhebok (*Pelea capreolus*)	16	7	6	13, 43
West Caucasian tur (*Capra caucasica caucasica*)	6	4	2	3
Zambesi lechwe (*Kobus leche leche*)	11	1	1	4
Zambian sable antelope (*Hippotragus niger kirkii*)	12	3	2	6
Zulu suni (*Neotragus moschatus zuluensis*)	5	0	0	.
**Family Cervidae**				
Bactrian wapiti (*Cervus elaphus bactrianus*)	1	0	0	.
Barasingha (*Cervus duvauceli branderi*)	3	2	1	23
Barbary red deer (*Cervus elaphus barbarus*)	13	1	1	4
Burmese thamin (*Rucervus eldii thamin*)	8	1	1	18
Calamian deer (*Axis calamianensis*)	8	0	0	.
European fallow deer (*Dama dama dama*)	2	1	0	.
Indian axis deer (*Axis axis*)	3	1	1	22
Indian hog deer (*Axis porcinus porcinus*)	15	1	1	24
Indian sambar (*Rusa unicolor unicolor*)	21	11	8	4, 10, 14, 21
Indochinese sika (*Cervus nippon pseudaxis*)	9	7	5	4
Javan rusa (*Rusa timorensis russa*)	28	15	14	4, 10, 17, 25, 37
Macneill's deer (*Cervus elaphus macneilli*)	2	0	0	.
Malayan sambar (*Rusa unicolor equina*)	6	4	4	4, 36
Mandarin sika (*Cervus nippon mandarinus*)	8	8	6	4
North indian muntjac (*Muntiacus muntjak vaginalis*)	1	1	1	9
Pere David's deer (*Elaphurus davidianus*)	3	1	1	4
Southern pudu (*Pudu puda*)	2	0	0	.
Western tufted deer (*Elaphodus cephalophus cephalophus*)	1	0	0	.
White-lipped deer (*Przewalskium albirostris*)	14	4	3	4, 7
**Family Giraffidae**				
Okapi (*Okapia johnstoni*)	5	1	0	.
Reticulated giraffe (*Giraffa camelopardalis reticulata*)	2	0	0	.
Uganda giraffe (*Giraffa camelopardalis rothschildi*)	3	0	0	.
**Family Moschidae**				
Siberian musk deer (*Moschus moschiferus moschiferus*)	3	0	0	.
**Totals**	715	210	161	44

^a^See Table 3 for a summary of viral genotypes.

All blood samples were processed for DNA extraction within 48 hours; all other samples were stored frozen at -20C or -80C until DNA extraction.

### DNA extractions

All DNA was extracted using the DNeasy blood and tissue kit (Qiagen, Valencia, California, USA) according to the manufacturer’s blood or tissue-sample protocol, except that the recommended amounts of sample were first placed with the lysis buffer in 1.5-ml screw-cap FastPrep vials containing ceramic beads, and lysed by agitation in a FastPrep shaker (Q-BIOgene, Carlsbad, Califonia, USA) at a speed of 4–5.5 for 40–60 sec, after which the lysate was transferred to a clean Eppendorf tube for continuation of the DNeasy protocol.

### Sanger sequencing

PCR using degenerate primers targeting a consensus region of the HV gene *DNA Polymerase* for amplification of all herpesviral DNA [40] was conducted to screen samples for herpesviruses. DNA (20–1000 nanograms) was added to a 25- or 50- μl reaction mixture containing 10mM Tris (pH 8.0), 50mM KCl, 5mM MgCl2, and 200μM each of dATP, dCTP, dGTP, and dTTP. AmpliTaq Gold DNA polymerase (Thermo Fisher Scientific, Carlsbad, California, USA) was used at a final concentration of 0.05 U/μl. Thermal cycling conditions were 9 min at 95°C, followed by 35 cycles of 94°C (30 s), 54°C (1 min), and 72°C (1 min), followed by a final extension at 72°C for 10 min. PCR products that were positive for HVs based on the consensus PCR (i.e. a band of expected size was present after electrophoresis) were purified and either direct-sequenced or cloned using the TOPO TA Cloning Kit (Invitrogen, Carlsbad California, USA). Sanger sequencing reactions were performed using the CEQ dye terminator cycle sequencing Quick-Start Kit (Beckman Coulter, Fullerton, California, USA). All Sanger sequences were acquired using a CEQ 2000XL capillary sequencer (Beckman Coulter). Sequence analysis and alignments were conducted using Geneious version 10.1.3 [43].

### Phylogenetic analysis

Phylogenetic analysis was performed using both nucleotide and translated amino acid sequence alignments. All alignments included *DPOL* reference sequences from viral species across multiple genera of *Alpha*-, *Beta*-, and *Gammaherpesvirinae* currently recognized by the ICTV [44]. All amino acid sequences were translated and aligned using MAFFT v7.388 [45], and a phylogenetic tree was constructed from the amino acid alignment (total alignment length: 71 amino acids) in MrBayes v 3.2.6 [46] with 4 heated chains of length 1 100 000, sampling every 200 iterations, and a burn-in of 100 000. The Jones-Taylor-Thornton model for amino acid evolution with gamma distributed rate heterogeneity (JTT +G) was selected for the amino acid analysis based on Akaike’s Information Criterion scores calculated by iModelGenerator v0.851 [47]. Maximum likelihood analysis was also performed using the amino acid alignment in the program PhyML v3.0 [48] with 1000 bootstrap replicates. The nucleotide sequences were aligned using CLUSTALW (total alignment length: 226bp) and phylogenetic analysis was repeated in MrBayes and PhyML using the same parameters used in the amino acid analysis. The General Time-Reversal model with a proportion of invariant sites and gamma distributed rate heterogeneity (GTR +I+G) model was selected as the optimal substitution model for both nucleotide analyses based on Akaike’s Information Criterion scores calculated in jModelTest v2.1.10 [49, 50]. All trees were visualized with TreeGraphv2.0 [51].

## Results

### Sanger sequencing

Among 715 animals tested, 210 (29.3%) were PCR positive but only 161 (22.5%) were PCR positive and confirmed with DNA sequencing (Table 1). The remaining 49 PCR positive cases did not produce an interpretable sequence after repeat attempts to sequence the PCR products. A complete list of the 161 PCR positive animals with confirmed HV sequencing and the samples tested is available in S1 Table. Eleven of the PCR positive animals were tested for HVs twice within a single year (Table 2). The HV sequences were assigned a numerical identification number and are referred to as *DPOL genotypes*. All *DPOL genotypes* and their translated amino acids were subjected to a nucleotide (Table 3) and protein (S2 Table) NCBI BLAST analysis to confirm viral identities. A complete list of host species for each *DPOL genotype* is included in S2 Table. In total, 44 *DPOL* genotypes were identified, including 10 identical to nucleotide sequences previously reported in GenBank. Of the 34 newly identified genotypes, 14 had greater than 95% nucleotide identity (range = 96.0–99.4%) to previously reported sequences, and 20 had less than 95% identity (range = 75.5–93.4%) to previously reported sequences (Table 3). Nucleotide distance values among *DPOL* genotypes ranged from 38.6% to 98.9% with a mean of 56.1%. For 26 sequences, nucleotide distances differed by less than 5%, and in 11 of those cases differed by only 1–3 base pairs (i.e. *DPOL* genotypes 11/12, 33/39, 44/45, 23/28, and 4/17/25). Notably, *DPOL 16* (143bp) was identical to both *DPOL 36* (173bp) and *DPOL* 19 (177bp); however, *DPOL 36* and *DPOL 19* were only 90.3% identical due to variation in the 5’ end of the sequence.

**Table 2:** Individual animals positive for herpesviruses and sampled at multiple time points.

Common name^a^	Animal ID	1st sample date (Month-Year)	Samples tested^b^	2nd sample date (Month-Year)	Samples tested^b^	*DPOL* genotypes detected
Mandarin sika	86	Oct-2006	BC, **N-SWAB**, O/P-SWAB	Sep-2007	**NT, RPLN, SP,** **TON**	4
Indochinese sika	106	Oct-2006	BC, **N-SWAB**, O/P-SWAB	Apr-2007	**BC,** NT, **RPLN,** SP, **TON**	4
Indian sambar	117	Nov-2006	BC, **N-SWAB**, O/P-SWAB	Jun-2007	BC, N-SWAB, **O/P-SWAB**	4, 10
Indian sambar	177	Nov-2006	**BC****, N-SWAB, O/P-SWAB**	Apr-2007	BC	14
Barbary red deer	154	Sep-2006	**BC****, N-SWAB**	Jul-2007	BC	4
Indian sambar	181	Dec-2006	**BC, N-SWAB, O/P-SWAB**	May-2007	BC	21
Slender-horned gazelle	174	Dec-2006	BC, **N-SWAB**, O/P-SWAB	Mar-2007	ABO, BC, N-SWAB, NT, O/P-SWAB, RPLN, SP, TON	15
Jimela topi	168	Oct-2006	BC, **N-SWAB**, O/P-SWAB	Mar-2007	BC, NT, RPLN, SP, TON	15
Addax	165	Nov-2006	BC, **N-SWAB**, O/P-SWAB	Aug-2007	BC	22
Jimela topi	26	Nov-2006	BC, N-SWAB, O/P-SWAB	Jan-2007	BC, **N-SWAB**, NT, O/P-SWAB	15
West caucasian tur	85	Oct-2006	BC, N-SWAB, O/P-SWAB	Sep-2007	NT, RPLN, SP, **TON**	3

BC, buffy coat; N-SWAB, nasal swab; O/P-SWAB oral/pharyngeal swab; NT, nasal tissue; RPLN retro pharyngeal lymph node; ABO abomasum

^a^ See Table 1 for genus and species names.

^b^ Samples in bold were PCR positive for herpesviruses (HVs) based on electrophoresis results, samples confirmed positive for HVs by DNA sequencing are underlined.

**Table 3:** Summary of *DPOL genotype* nucleotide BLAST results.

Genotype designation in current study	Identity	Genbank taxonomy (accession number)^a^	Provisional species name for new virus (host common name)	Sequence Length (bp)	Genbank numbers
1	75.60%	Vaal Rhebok HV (AY664876)	*Philantomba monticola* (Cape blue duiker) GHV-1	184	MN599417
2	99.40%	Fallow Deer LHV (DQ083951)	Bovidae GHV-1	174	MN599418
3	100%	West Caucasian tur macavirus (HM216457)	.	177	HM216457
4	100%	Elk GHV (KY612411)	.	174	KY612411
5	76.20%	AlHV-1 (AB194010)	Bovidae GHV-2	174	MN599419
6	83.90%	AlHV-1 (MG000864)	Bovidae GHV-3	174	MN599420
7	97.70%	Nubian ibex RHV (AY212112)	Ruminantia GHV-1	177	MN599421
8	98.90%	OvHV-2 (HQ450395)	*Eudorcas rufifrons* (Sudan red-fronted gazelle) GHV-3	177	MN599422
9	82.90%	Elaphurus davidianus GHV (KY621347)	Ruminantia GHV-2	174	MN599423
10	99.30%	Waterbuck macavirus (HM216473)	Ruminantia GHV-3	174	MN599424
11	88.40%	Hexaprotodon liberienes GHV-1 (AY197559)	*Cephalophinae* GHV-1	174	MN599425
12	88.40%	Hexaprotodon liberienes GHV-1 (AY197559)	*Cephalophus rufilatus* (Red-flanked duiker) GHV-1	174	MN599426
13	100%	Vaal Rhebok HV (AY664876)	.	174	AY664876
14	96%	Sambar GHV isolate (KY612408)	*Rusa unicolor unicolor* (Indian sambar) GHV-1	174	MN599427
15	87.90%	AlHV-2 (KF274499)	Bovidae GHV-4	174	MN599428
16	77.40%	SpHV-1 (AB194011)	Bovidae GHV-5	143	MN599429
17	99.40%	Elk GHV isolate (KY612412)	*Rusa timorensis* (Javan rusa) GHV-1	175	MN599430
18	96.80%	West Caucasian tur macavirus (HM216457)	*Rucervus eldii* (Burmese thamin) GHV-1	177	MN599431
19	77.40%	SpHV-2 (AB194011)	Bovidae GHV-9	177	MN599432
20	87.80%	Al HV-2 (KF274499)	*Damaliscus korrigum* (Jimela topi) GHV-1	176	MN599433
21	96%	Sambar GHV isolate (KY612408)	*Rusa unicolor* unicolor (Indian sambar) GHV-2	173	MN599434
22	93.40%	Waterbuck macavirus (HM216473)	Ruminantia GHV 4 (waterbuck-like)	174	MN599435
23	100%	SpHV-1 (AB194010)	.	174	AB194010
24	82.70%	Pere david's deer GHV isolate (KY621347)	*Axis porcinus* (Indian hog deer) GHV-1	174	MN599436
25	100%	Elk GHV isolate (KY612412)	.	174	KY612412
26	100%	Impala HV-1 (AB194012)	.	175	AB194012
27	99.40%	Addax RHV (AY237366)	*Hippotraginae* GHV-2	175	MN599437
28	98.30%	SpHV-1 (AB194010)	*Capra nubiana* (Nubian ibex) GHV-1	176	MN599438
29	82.90%	SpHV-1 (AB194010)	*Gazella subgutturosa* (Persian goitered-gazelle) GHV-1	180	MN599439
30	77.90%	SpHV-2 (AB194011)	*Eudorcas rufifrons* (Sudan red-fronted gazelle) GHV-1	174	MN599440
31	100%	Oryx RHV-1 (AY212113)	.	174	AY212113
32	100%	Bovine HV-6 (KJ705001)	.	174	KJ705001
33	76.20%	SpHV-2(AB194011)	*Eudorcas rufifrons* (Sudan red-fronted gazelle) GHV-2	174	MN599441
34	97.50%	Waterbuck macavirus (HM216473)	*Kobus ellipsiprymnus* (Ellipsen waterbuck) GHV-1	165	MN599442
35	98.60%	Waterbuck macavirus (HM216473)	*Kobus ellipsiprymnus* (Ellipsen waterbuck) GHV-2	180	MN599443
36	77.40%	SpHV-2 (AB194011)	*Rusa unicolor equina* (Malayan sambar) GHV-1	173	MN599444
37	97.50%	Elk GHV isolate (KY612412)	*Rusa timorensis* (Javan rusa) GHV-2	164	MN599445
38	100%	CaHV-2 (HM216474)	.	177	HM216474
39	76.90%	SpHV-2 (AB194011)	*Madoqua kirkii* (Cavendish's dik dik) GHV-1	174	MN599446
40	97.70%	SpHV-1 (AB194010)	*Gazella spekei* (Speke's gazelle) GHV-1	173	MN599447
41	69.90%	Vaal Rhebok HV (AY664876)	*Philantomba monticola* (Cape blue duiker) GHV-2	174	MN599448
42	75.50%	SpHV-2 (AB194011)	*Litocranius walleri* (Southern gerenuk) GHV-1	174	MN599449
43	89.40%	Wasserbock macavirus (HM216473)	*Pelea capreolus* (Vaal rhebok) GHV-1	143	MN599450
44	100%	OvHV-2 (HQ450395)	.	177	HQ450395

HV, herpesvirus; LHV, lymphotrophic HV; RHV, rhadinovirus; GHV, gammaherpesvirus; OvHV-2, Ovine HV-2; CaHV-2, Caprine HV-2; AlHV-1, Alcelaphine HV-1; AlHV-2, Alcelaphine HV-2; SpHV-1, Springbok HV-1; SpHv-2, Springbok HV-2.

^a^BLAST data accessioned on 10/21/2019

The 34 *DPOL genotypes* with less than 100% similarity to previously reported viruses in GenBank were assigned provisional names after their host species and tentatively assigned to *Gammaherpesvirinae* based on our phylogenetic and BLAST analysis; however, the primary host of these putative viruses remains to be confirmed. A single genotype identified in multiple hosts from the same sub-family or family was named after the sub-family or family; alternatively, a genotype identified in multiple hosts from different families within the order Ruminantia was assigned the name “Ruminantia”. All *DPOL genotypes* are listed in Table 2 with their corresponding GenBank accession numbers.

### Phylogenetic analyses

**Fig 1:**
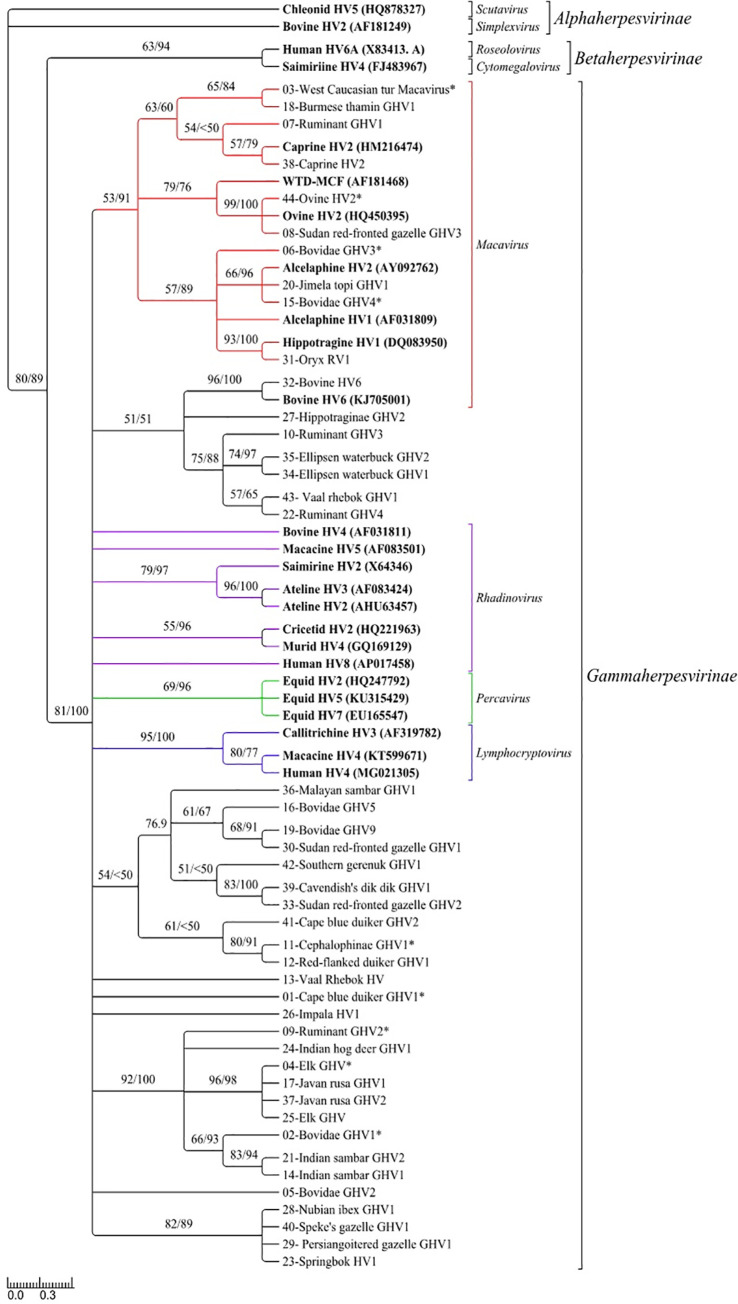
Phylogenetic relationships of translated amino acid sequences from *DPOL genotypes* detected in ruminant species from the San Diego Zoo or it's Safari Park using PCR. The unrooted phylogenetic tree was constructed using translated amino acid sequences (71 aa) from *DPOL* genotypes in Table 3 and herpesviruses from GenBank. Accession numbers for each reference sequence are included in the figure. Maximum-likelihood bootstrap values are to the left of the slash ("/") and Bayesian Posterior Probabilities, expressed as a percentage, are to the right at each node. Nodes with Maximum-likelihood bootstrap values less than 50 were collapsed. The numerical *DPOL* genotype is followed by the provisional common name for new viral species or the previously described virus name from Table 3. *DPOL genotypes* obtained from clinical MCF cases are indicated with an asterisk (*), and references from GHV sub-families recognized by the International Committee on the Taxonomy of Viruses are indicated in bold. Known genera are indicated by color: red (*Macavirus*), purple (*Rhadinovirus*), blue (*Lymphocryptovirus*), and green (*Percavirus*). Known genus and sub-families are also indicated with brackets.

The phylogenetic trees created by MrBayes and PhyML were similar for both amino acid (Fig 1) and nucleotide (S1 Fig) analyses and support the placement of all *DPOL* genotypes described here within *Gammaherpesvirinae*. Our phylogenetic analyses also support the presence of multiple lineages within *Gammaherpesvirinae* [52], including the genera *Macavirus*, *Rhadinovirus*, *Lymphocryptovirus*, and *Percavirus*. Many of the nodes within *Gammaherpesvirinae* strongly supported by Bayesian analysis lacked high support from maximum likelihood analysis; therefore, the more conservative maximum likelihood tree is shown for both amino acid (Fig 1) and nucleotide (S1 Fig) analyses with bootstrap and posterior probability values for each node.

### MCF cases

A total of nine GHVs were detected in the 11 clinical MCF cases included in our sample population (Table 4). Six of the GHVs detected in the clinical MCF cases were also detected in healthy individuals with no clinical signs of disease. Phylogenetic analyses revealed that only four of the nine GHVs isolated from clinical MCF cases grouped within the *Macavirus* genus (Fig 1). The remaining five GHVs isolated from clinical MCF cases could not be categorized into any single GHV genus, respectively.

**Table 4:** Summary of PCR and sequencing results from individual animals with clinical signs of MCF.

Common name^a^	Animal ID	Genotype designation in present study	Viral species name (accession number)
Cape blue duiker	100	1	Cape blue duiker GHV-1
Cape blue duiker	116	1, 2	Cape blue duiker GHV-1, Bovidae GHV-1
Cape blue duiker	121	11	Duiker GHV-1
Domestic goat	112	44	Ovine herpesvirus 2 (HQ450395)
Eastern bongo	113	3	West Caucasian tur macavirus (HM216457)
Eastern bongo	122	3	West Caucasian tur macavirus (HM216457)
Eastern bongo	123	3	West Caucasian tur macavirus (HM216457)
Eastern yellow-backed duiker	111	6	Bovidae GHV-3
Eastern yellow-backed duiker	119	4, 9	Elk GHV (KY612412), Ruminantia GHV-2
Red-flanked duiker	120	11	Duiker GHV-1
Slender-horned gazelle	124	15	Bovidae GHV-4

GHV, gammaherpesvirus

^a^See Table 1 for genus and species names.

## Discussion

Our study presents a broad survey of the genetic variation of GHVs within ruminant species in the zoological collections of two facilities. Nearly 30% of 715 opportunistically tested individuals of 96 species at the San Diego Zoo and the San Diego Zoo Safari Park tested positive for GHVs of which only 11 (6.83%) showed clinical signs of MCF disease. Based on Sanger sequencing results of a subset (76.7%) of positive cases, our data confirm the presence of 10 previously discovered GHVs (including BoHV-6 (KJ705001), CaHV-2 (HM216474), Elk GHV (KY612412), Elk GHV (KY612411), Impala HV-1 (AB194012), Oryx rhadinovirus 1 (AY212113), OvHV-2 (HQ450395), Springbok HV-1 (AB194010), Vaal Rhebok HV (AY664876), West Caucasian tur macavirus (HM216457)), and identify 34 novel GHV genotypes in ruminants. This supports many previous observations that GHVs are widespread in asymptomatic hosts and suggests that the potential diversity and host ranges of GHVs are greater than previously known [53].

Our phylogenetic analyses of both nucleotide and translated amino acid data indicate that the novel viral genotypes were members of the family *Gammaherpesvirinae*; however, the trees also revealed many unresolved polytomies. We attribute the unresolved branch orders within our phylogenetic analysis to the relatively short alignment length of our dataset and suggest that longer DNA sequences be used to resolve the phylogenic relationships in future analyses. Previous studies of GHV phylogeny using longer DNA segments (>3kb) and a multi-gene approach similarly observed polytomies within *Gammaherpevirinae* clades and also suggest that these polytomies could likely be resolved using even more extensive sequence data [52]. Despite the use of a relatively small DNA sequence fragment there are some strongly supported (i.e. bootstrap and posterior probability >70) clusters within the tree. The high nucleotide identities and phylogenetic confidence among the members of these clusters suggests that many of the GHVs in our sample population may be unique genotypes of the same viral species. However, more data is needed to characterize these putative viral sequences as unique species and confirm their assignment to known genera.

Co-infection was identified in five out of 161 individuals. In three of those animals (Animal ID: 18, 118, and 119), two GHVs were identified in a single tissue, blood, or swab sample. Co-infection of an individual host with multiple HVs has been documented previously [9, 54–56]; therefore, it was not surprising to identify multiple HVs within a single host species or individual animal from our sample population, especially given that many of the species had not been previously screened for HVs.

Seventeen of the 44 GHVs were identified in more than one species (S2 Table), with four GHVs observed in host species across both families *Cervidae* and *Bovidae*. The three species with the highest number of PCR positive individuals sampled [i.e. Ellipsen waterbuck (*Kobus ellipsiprymnus ellipsiprymnus*), Javan rusa (*Rusa timorensis russa*), and Indian sambar (*Rusa unicolor unicolor*)] also had the highest number of unique GHVs. The three genotypes identified in Ellipsen waterbuck (i.e. *DPOL genotypes* 10, 34, 35) shared 86.8–92.0% nucleotide identity and all clustered together with high support in the phylogenetic analysis (Fig 1). Two of three Ellipsen waterbuck genotypes were only observed in Ellipsen waterbuck (i.e. *DPOL genotype* 34 and 35), while the third (*DPOL genotype* 10) was observed in multiple species, including Javan rusa and Indian sambar. The remaining four genotypes observed in Javan rusa (i.e. *DPOL genotypes* 4, 17, 25, 37) were highly similar (98.3–96.3% nucleotide identity) and clustered together in the phylogenetic analysis with high support. However, Ruminant GHV-3 (*DPOL* genotype 10) shared only 54.8–57.6% nucleotide identity with the other four Javan rusa *DPOL* genotypes, and phylogenetic analysis clustered it with other genotypes mostly observed in members of the family *Bovidae*. The surveyed Indian sambar followed a similar trend with the majority of individuals (7 out of 8 total) infected with two highly similar (97.1% nucleotide identity) GHVs (*DPOL* genotype 14 and 21), and a single individual infected with *DPOL* genotype 10. The presence of multiple highly related viruses in a single host species was not surprising given that (1) many of these species have not been surveyed for GHVs previously, and (2) the surveyed population includes animals bred in zoos rather than wild populations. Initial investigations of GHV evolutionary relationships proposed host-adaptation through the co-speciation of host and virus with limited inter-species transfer of viruses [2]; however, later investigations found that many of the GHV lineages were not compatible with a single co-speciational scheme [52]. Co-speciation and duplication events may explain broad evolutionary patterns within the *Herpesviridae* family as previously suggested [2, 57] but the unpredicted complexities of the phylogenetic signal observed in our data could be the result of co-housing animals from geographically separate populations. Thus, the genetic diversity observed within our dataset may be the product of viral host-switching or spillover events from reservoir species to novel hosts given the close proximity of diverse host species in a zoological setting. Animals in this study were not all housed together at the same time, and closer scrutiny of complex individual movement patterns through shared environments may further reveal natural carriers and aberrant hosts. Additionally, future efforts should be aimed at whole genome sequencing in order to tease apart the complex web of evolutionary process shaping GHV diversity and disease processes.

The data further expands the host range of many previously described viruses, including the West Caucasian tur macavirus. This virus was previously identified in West Caucasian tur and its host range may now include four additional species. Our data also suggests that this virus may be associated with MCF in Eastern bongos which are listed as a critically endangered species by the International Union for the Conservation of Nature [58]. Three out of four Eastern bongos sampled had clinical signs of MCF and were infected with West Caucasian tur macavirus. The remaining Eastern bongo was sampled two years after the diseased bongos, and HVs were not detected in the sample at that time. Surprisingly, the *Macavirus* OvHV-2 was detected in a single domestic goat with clinical signs of MCF. A previous study indicated goats as subclinical carriers of OvHV-2 [59]; however, a recent study also identified MCF in a domestic goat infected with OvHV-2 [17]. Overall, the presence of MCF-related viruses in both healthy and diseased animals of the same species suggests a complex relationship between the virus and host.

Multiple factors that may have influenced viral detection in our study, including but not limited to the intermittence of herpesviral shedding and/or viremia in our study population, and the viral detection limit of the PCR assay. Previous investigations have suggested age, season, and genetic background of the infected individual as potential variables affecting viral shedding and viremia of GHVs [60, 61]. Eleven of the 168 individuals with confirmed GHVs by Sanger sequencing were sampled at multiple time points within a single year, including three individuals that were positive at both time points, six that were only positive during the first time point, and two that were only positive during the second time point (Table 2). In one of the animals that was positive during both sampling events, Indian sambar (*Rusa unicolor unicolor*) #117, the consensus based PCR and Sanger sequencing identified two unique genotypes. In 2006 the nasal swab was positive for the Elk gammaherpesvirus (Genbank# KY612412), and in 2007 the oral/pharyngeal swab was positive for Ruminantia gammaherpesvirus 3 (Genbank# MN599424). Blood samples (Buffy coat) were also tested at both time points in nine of the 11 samples. The blood samples of four individuals were PCR positive for GHVs, but none of them were positive at both time points. These results may be explained by a seasonal influence or other factors that may affect viral shedding and viremia of the viruses detected in these samples. Although, it remains possible that the virus was either not sampled due to sampling error, recently contracted, or present at undetectable levels during the negative sampling events. More research is needed to determine the pathogenesis of the newly discovered viruses as well as the viral detection limit of the PCR primers used in this study. Therefore, the viruses identified in this study are presented as a conservative number of viral species present within a single sample at a single time point.

In summary, the GHV subfamily is an extremely diverse collection with members that have the ability to infect multiple species, and our data provides a snapshot in time of the complex genetic diversity within our sample population. The reported diversity of GHVs increases with sampling of more ruminants, and this diversity may reflect the evolutionary capacity of HVs to adapt to new and varied host conditions. While this study represents the broadest survey of GHV diversity within ruminant species to date, more work is required to define the host-virus relationship and disease complex of the novel viruses presented. A particularly exciting potential for future investigations is correlative studies looking for the existence of one or several common virulence markers that could be targeted in new therapies or vaccines. Future studies may also be directed at sampling wild populations of the species investigated in our study to determine if the viral sequences obtained from animals housed in a zoo reflect the diversity of viruses present in free-ranging populations.

## Supporting information

S1 Table Sample summary of positive herpesviral PCR and Sanger sequencing results. (XLSX)

S2 Table Amino acid GenBank BLAST results. (XLSX)

S1 Fig Phylogenetic relationships of *DPOL* genotypes detected in ruminant species from the San Diego Zoo or San Diego Zoo Safari Park using PCR, including a selection of sequences from multiple genera of Alpha-, Beta-, and Gammaherpesvirinae. (TIF)
