# Supplementary figures and images for: Herpesvirus surveillance and discovery in zoo-housed ruminants
Source: PLoS One. 2021 Jan 28;16(1):e0246162. doi: 10.1371/journal.pone.0246162 (PMC7842878; doi:10.1371/journal.pone.0246162)

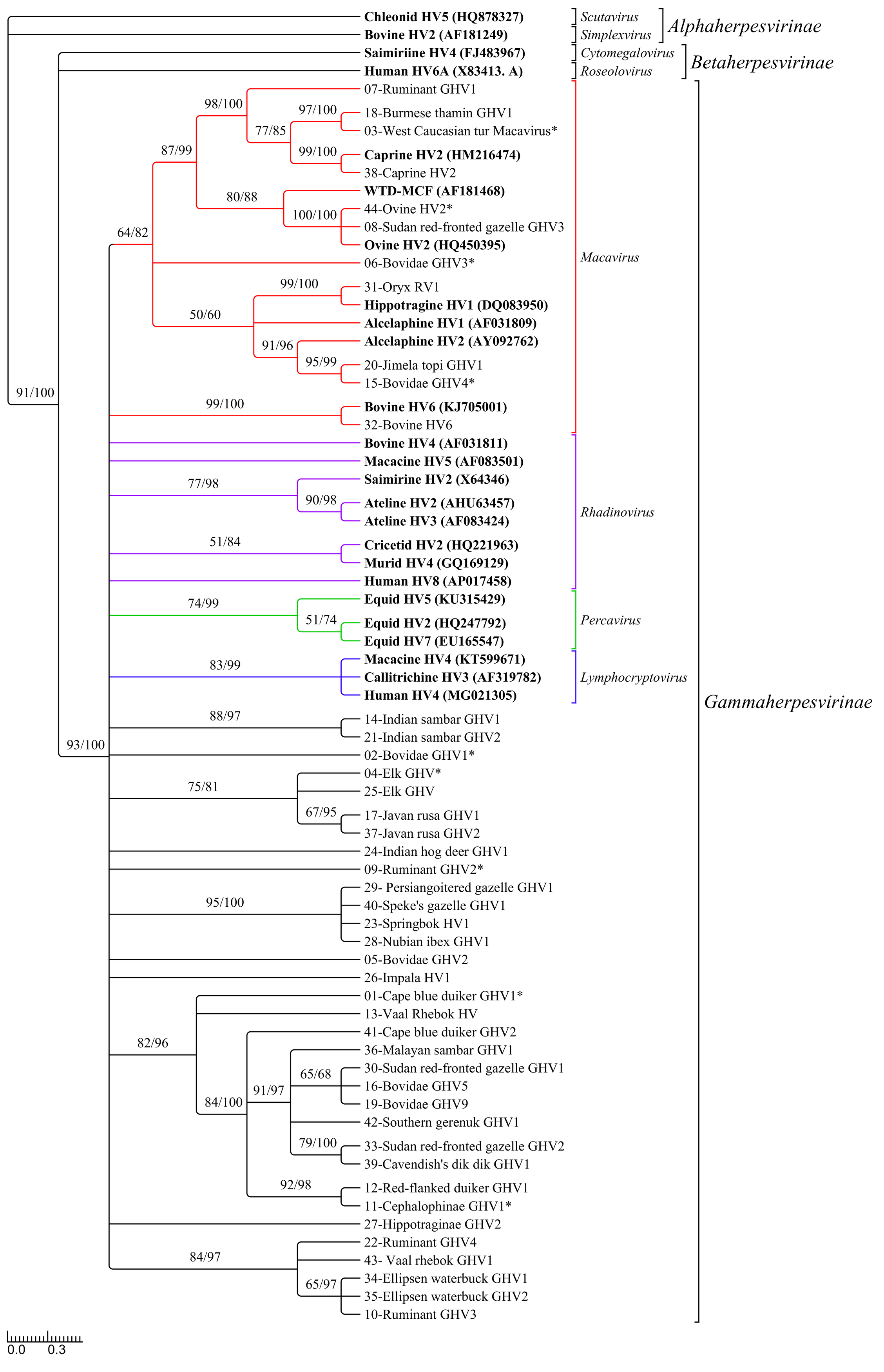

Supplement: S1 Fig — (TIF) [file pone.0246162.s003.tif]
